# Advances on the Role and Applications of Interleukin-1 in Tuberculosis

**DOI:** 10.1128/mBio.03134-21

**Published:** 2021-11-23

**Authors:** Diogo Silvério, Rute Gonçalves, Rui Appelberg, Margarida Saraiva

**Affiliations:** a i3S-Instituto de Investigação e Inovação em Saúde, University of Porto, Porto, Portugal; b IBMC-Instituto de Biologia Molecular e Celular, University of Porto, Porto, Portugal; c Doctoral Program in Molecular and Cell Biology, ICBAS-Instituto de Ciências Biomédicas Abel Salazar, University of Porto, Porto, Portugal; d Master’s Program in Cell and Molecular Biology, FCUP-Faculdade de Ciências, University of Porto, Porto, Portugal; e ICBAS-Instituto de Ciências Biomédicas Abel Salazar, University of Porto, Porto, Portugal; Ohio State University; Ohio State University

**Keywords:** host-pathogen interactions, immune response, infectious disease, interleukins, tuberculosis

## Abstract

Interleukin-1 (IL-1) is a key player in the immune response to pathogens due to its role in promoting inflammation and recruiting immune cells to the site of infection. In tuberculosis (TB), tight regulation of IL-1 responses is critical to ensure host resistance to infection while preventing immune pathology. In the mouse model of Mycobacterium tuberculosis infection, both IL-1 absence and overproduction result in exacerbated disease and mortality. In humans, several polymorphisms in the *IL1B* gene have been associated with increased susceptibility to TB. Importantly, M. tuberculosis itself has evolved several strategies to manipulate and regulate host IL-1 responses for its own benefit. Given all this, IL-1 appears as a promising target for host-directed therapies in TB. However, for that to succeed, more detailed knowledge on the biology and mechanisms of action of IL-1 *in vivo*, together with a deep understanding of how host-M. tuberculosis interactions modulate IL-1, is required. Here, we discuss the most recent advances in the biology and therapeutic potential of IL-1 in TB as well as the outstanding questions that remain to be answered.

## INTRODUCTION

Interleukin-1 (IL-1) was described almost 80 years ago, by Menkin, as a factor mediating tissue injury due to inflammation (1). At the time, this factor was termed “leukocytic pyrogen.” Thirty years later, this pyrogen protein was shown to induce the activation and proliferation of lymphocytes (2). Upon the introduction of the term “interleukin” in 1979, the leukocytic pyrogen was coined “IL-1” (3). Two agonist molecules with IL-1 biological activity were later discovered and named IL-1α and IL-1β (4). The discovery of other related molecules (IL-18, IL-33, IL-36, and IL-38) led to the definition of the IL-1 superfamily (5). Members of this superfamily exert their function by binding to one of the several members of the IL-1 family of receptors and coreceptors (6). Both IL-1α and IL-1β (collectively referred to as IL-1) exert their activity by binding IL-1 receptor 1 (IL-1R1) (6). Interestingly, the antagonist molecule (IL-1RA) also binds to this receptor, preventing the binding of IL-1 molecules and consequently blocking their biological effects (6).

IL-1 plays many different roles, from mediating the immune response to infection (7) to regulating vascular permeability and angiogenesis (8). Deregulated IL-1 responses have been associated with the development and progression of cancer (9) and also with autoimmune diseases such as rheumatoid arthritis (10). These findings eventually led to the development of anakinra, a drug that mimics IL-1RA, thus blocking IL-1 responses (11). Anakinra signals the potential of IL-1 modulation to treat disease as it proved effective in the case of rheumatoid arthritis and other acute or chronic autoinflammatory conditions (11). Nevertheless, the administration of anakinra may result in an increased risk of infections, including tuberculosis (TB) (12–14).

TB is a respiratory disease that has afflicted humans since primordial times. Despite the decrease in TB incidence rates in the 21st century, about 10 million new TB cases and 1.4 million TB-related deaths were registered in 2019 (15). Furthermore, one-fourth of the human population is estimated to be latently infected with Mycobacterium tuberculosis, a fraction of whom will develop TB (15). The immune response to M. tuberculosis and the role played by various cytokines have been extensively reviewed elsewhere (16–18). Within the IL-1 family of cytokines, IL-1α, IL-1β, and IL-1RA have been the most studied in TB (16, 17). In this review, we focus on the protective and regulatory effects of IL-1 during TB, how IL-1 is regulated by the interaction of M. tuberculosis with host cells, and the therapeutic/diagnostic promise of IL-1.

## IL-1 PLAYS (MOSTLY) A PROTECTIVE ROLE IN TB

Several studies were performed in cohorts of TB patients with the aim of defining the contribution of IL-1 to TB protection/susceptibility. The genetic variants −511(T/C) and +3953(T/C) of the *IL1B* gene have been addressed in multiple studies, but various associations with TB susceptibility have been described (19–26). As an example, the T allele in position −511, which is functionally related to lower-level IL-1β production by stimulated cells, has been associated with increased TB susceptibility in a Gambian population, whereas in the same study, no association was found for polymorphisms in the +3953 position (20). However, in another study, involving a Colombian population, the very opposite findings were reported, with the +3953 T allele being associated with low IL-1β expression levels and conferring TB protection (21). Furthermore, the high-IL-1β-producing T allele in promoter position −31 was shown to be associated with an increased risk of active TB and poor clinical outcomes, likely due to increased neutrophil infiltration (24). The observed discrepancies may reflect variable numbers of patients as well as the genetic makeup of both the host and the pathogen populations in the study cohorts. Additionally, different populations are subjected to distinct infection pressures, which may influence transmission rates, doses of infection, and outcomes of TB. These variations may in turn interfere with a protective or detrimental role for IL-1 in TB. Therefore, collectively, the findings coming from genetic studies highlight the importance of tight regulation of IL-1β during TB. This is further supported by observations coming from the clinical manipulation of IL-1β. As mentioned above, therapeutic blockade of IL-1 with anakinra has been associated with the risk of TB, supporting a protective role of IL-1 in TB (13, 14). However, increased levels of IL-1β and ratios of IL-1β/IL-1RA were shown to be associated with tissue necrosis and cavity formation in TB patients (27).

Studies performed in the mouse model of infection unequivocally illustrate the importance of IL-1 for host defense against TB. M. tuberculosis infection of genetically engineered mouse models with abrogated synthesis of IL-1α and IL-1β or their target receptor IL-1R1 revealed high mortality rates, accompanied by increased bacterial burdens and extensive pathology in the lungs (28–34). Furthermore, abrogation of the adaptor molecule myeloid differentiation factor 88 (MyD88) resulted in high susceptibility to experimental infection and premature death (35–37), which did not result from defective Toll-like receptor (TLR) signaling but instead resulted from defective IL-1R signaling (32, 38). Although the protection afforded by a competent IL-1R in experimental M. tuberculosis infection is indisputable, whether the determinant protective role is played by IL-1α or IL-1β is still a matter of debate. Mice lacking IL-1β displayed acute mortality upon M. tuberculosis infection, similarly to IL-1R1-deficient mice, in support of a major role played by IL-1β (32). However, in other studies, genetic deficiency or neutralization of IL-1α, but not of IL-1β, conferred higher susceptibility to infection (29, 39). Moreover, while IL-1α and IL-1β double deficiency consistently recapitulated the phenotype of IL-1R1-deficient mice, the presence of either cytokine ensured bacterial burden and pathology control (40), thus suggesting a certain degree of redundancy. Although these various studies use similar mouse (C57BL/6) and M. tuberculosis (H37Rv strain) genetic backgrounds, there are some experimental differences that may account for the distinct results: the infection route (aerosol versus intranasal), the dose of bacteria in infection (low versus high), the time points chosen for the analyses, the inactivating mutations in the *Il1b* gene, and the use of genetic abrogation versus antibody neutralization. Furthermore, it is possible that imbalances in the animal microbiome may explain some current contradictions, given the emerging evidence for cross talk between the microbiota and TB outcomes (41). Of note, the reasons underlying discrepant IL-1 impacts on TB in human and mouse studies are therefore parallel. Different doses of bacteria used in experimental infections may relate to differences in infection pressures in human populations, data collected at different time points *in vivo* may reflect differences across the spectrum of disease in human TB, and an influence of the microbiome is likely to occur in mice and humans alike, especially considering the diverse environments and life qualities that different populations experience around the globe. Thus, although the interpretation of the role of IL-1 in TB needs to be taken with care, the parallel study of human and mouse data as well as the modulation of the above-discussed variables in experimental models are opening very challenging and promising avenues in TB research.

As discussed above, the protective role of IL-1 in TB seems to be a dynamic one. In both nonhuman primates and a susceptible mouse model, the administration of anakinra 2 weeks after M. tuberculosis infection, as an adjunct to linezolid treatment, proved beneficial in controlling inflammation and lung damage (42). Thus, whereas initial IL-1 responses may be critical for protection, at a later stage, they may become detrimental and contribute to tissue damage. It is tempting to speculate that this dynamic role, leading to protective or detrimental effects of IL-1, results from changes in the cellular and molecular microenvironments. During early time points postinfection, IL-1 would mainly affect stromal and innate immune cells, possibly activating protective mechanisms. Later on, the establishment of acquired immune responses induces a dramatic change in the lung microenvironment, with IL-1 potentially acting on different cell types and possibly controlling different steps of the immune response. The tuning of these different steps is likely set by the characteristics of the host, the infecting M. tuberculosis strain, as well as the doses of infection. Importantly, a dynamic role of IL-1 has been also demonstrated in other infectious diseases, where excessive production leads to inflammatory damage, but too little of this cytokine is insufficient to trigger an immune response to fight off the pathogen (43, 44).

## CELLULAR SOURCES AND CELLULAR TARGETS OF IL-1 IN TB

Innate immune cells are described as the main *in vivo* cellular sources of IL-1β during experimental M. tuberculosis infection. Inflammatory monocytes, macrophages, and dendritic cells all upregulate their production of IL-1 upon M. tuberculosis uptake in the lung (16). Neutrophils are also recruited to the lung and take up M. tuberculosis, but their contribution to IL-1 production seems to be much lower (16). Interestingly, lung-residing myeloid-derived suppressive cells have also been described to release IL-1β during M. tuberculosis infection despite maintaining their suppressive activity (45). More recently, alveolar macrophages were also found to produce IL-1β *in vivo* upon infection with M. tuberculosis (46). These findings are paralleled *in vitro* as the secretion of IL-1α and -β (and other cytokines) was reported in a model of M. tuberculosis-infected alveolar macrophages (47). The molecular mechanisms regulating the production of IL-1 by innate immune cells are discussed in more detail in the next section.

Myeloid cells are also well described as cellular targets of IL-1 during TB. The functional consequences of IL-1R activation in myeloid cells are mainly studied *in vitro* and suggest that IL-1 triggers several microbicidal mechanisms, thus offering a potential explanation for the protective role of this cytokine during infection. Among the described microbicidal mechanisms are the induction of autophagy in the macrophage cell line Raw264.7 (48) and the potentiation of the production of tumor necrosis factor (TNF), through a caspase-3 (CASP-3)-dependent mechanism, in both human- and murine-derived macrophages (49). Without the presence of a functional IL-1R, mouse macrophages are more easily infected by M. tuberculosis, hinting at a further role for this molecule in preventing bacterial dissemination (28). Furthermore, IL-1 appears to instruct the type of cell death upon phagocytosis of bacteria by enhancing the production of prostaglandins, particularly prostaglandin E_2_ (PGE_2_) (31). Low-level prostaglandin production has been described to drive infected macrophages into necrotic rather than apoptotic cell death, favoring the spread of M. tuberculosis within the host (50). The cross talk between IL-1 and the production of PGE_2_ is also evident in human macrophages and *in vivo*, as the blocking of IL-1 or IL-1R results in lower-level production of PGE_2_ in mice, culminating in the poor control of M. tuberculosis (31).

A growing body of evidence highlights an important role for nonhematopoietic cells as *in vivo* targets of IL-1. The course of M. tuberculosis infection has been assessed in bone marrow chimeric mice lacking a competent IL-1R in hematopoietic or nonhematopoietic compartments. In one study, deficiency of IL-1R in hematopoietic cells resulted in high susceptibility to infection, with mice succumbing to disease similarly to fully IL-1R-deficient mice (29), therefore suggesting that immune cells are the main targets of IL-1 during M. tuberculosis infection. However, another study reported that IL-1R signaling in hematopoietic or nonhematopoietic cells was sufficient to afford a degree of protection similar to that seen in wild-type (WT) mice (28). This conclusion was further reinforced by the observation that CD45-restricted IL-1R-deficient mice did not show increased susceptibility to M. tuberculosis infection (28). Since in these two studies (28, 29), the experimental setups were similar, with equivalent mouse and bacterial genetic backgrounds as well as routes and doses of administration, further studies are required to unlock the cellular targets of IL-1 during M. tuberculosis infections. For this, the study of infection in hematopoietic versus nonhematopoietic cell-restricted IL-1R-deficient mice and detailed analysis of nonhematopoietic cells upon *in vivo* infection would be important.

More recently, the migration of M. tuberculosis-infected alveolar macrophages into the lung interstitium was shown to be dependent on IL-1R signaling triggering in lung epithelial cells (46). Also, in an *in vitro* coculture system of macrophages and small airway epithelial cells, macrophages produced IL-1β in response to M. tuberculosis infection, but this IL-1β acted on the epithelial cells, inducing their production of the antimicrobial peptide DEFB4/HBD2, which was effective in controlling M. tuberculosis replication in these cells (51). The fact that IL-1 increases the expression of adhesion molecules on endothelial cells (52, 53), in this way promoting the migration of immune cells from the vasculature to the tissue, may also uncover a potential role of endothelial cells as targets for IL-1 in TB. Collectively, these findings hint at a role played by IL-1R signaling not only in hematopoietic but also in stromal compartments during the establishment of TB, as is the case for other lung infections (54, 55). Consequently, it is possible that the *in vivo* role of IL-1 is not limited to the activation of microbicidal mechanisms focused on pathogen elimination but instead also includes the modulation of the immune response in a more holistic manner. Analysis of infection progression together with a deep characterization of the local immune response triggered in cell-restricted models of IL-1R deficiency will be important to clarify the role of IL-1 in TB.

## REGULATION AND CROSS-REGULATION OF IL-1 DURING M. TUBERCULOSIS INFECTION

The production of IL-1β by myeloid cells is controlled following a two-step model that involves regulation at the transcriptional and posttranslational levels (7). Briefly, the transcription of the *Il1b* gene depends on the activation of pattern recognition receptors (PRRs) in myeloid cells and the triggering of specific intracellular signaling cascades and transcription factors (7). This first step results in the production of an inactive form of IL-1β (pro-IL-1β). A second step, consisting of the assembly of inflammasome components and the enzymatic action of caspases (CASPs) (7), is then required to cleave pro-IL-1β and promote the secretion of the bioactive molecule IL-1β.

**FIG 1:**
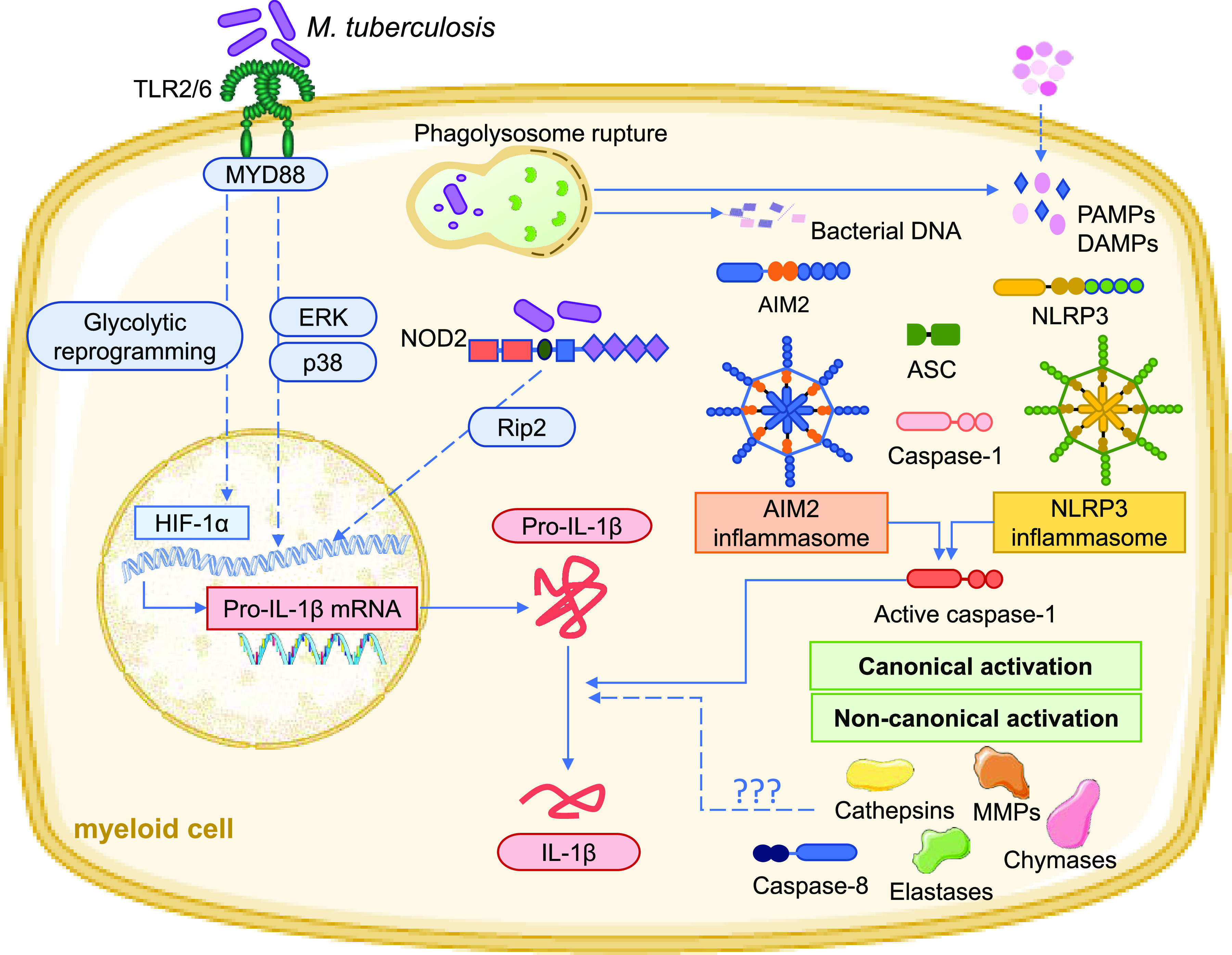
Molecular mechanisms leading to IL-1β production in M. tuberculosis-infected cells. The recognition of M. tuberculosis molecular patterns by TLR2/6 or NOD2 induces a series of signaling cascades that culminate in the transcription of the IL-1β mRNA. The glycolytic reprogramming of the infected macrophage also enhances *Il1b* transcription. Biological activation of IL-1β requires cleavage of pro-IL-1β through canonical or noncanonical mechanisms. Canonical activation consists of the assembly of NLRP3 and AIM2 inflammasomes, which are triggered by the recognition of pathogen-associated molecular patterns/damage associated molecular patterns (PAMPs/DAMPs) and bacterial DNA, respectively, resulting from the export of bacterial products from the phagolysosome. The assembly of the inflammasomes leads to the recruitment of CASP1 by ASC. CASP1 becomes activated and cleaves pro-IL-1β into active IL-1β. Noncanonical activation is much less studied in the context of M. tuberculosis but may be mediated by elastases, matrix metalloproteinases (MMPs), other caspases, and chymases.

In the context of M. tuberculosis infection, the transcription of the IL-1β-encoding gene is induced in human peripheral blood mononuclear cells (PBMCs) and human and mouse macrophages through the activation of pathways downstream of TLR2/TLR6 and NOD2 receptors (56) (Fig. 1). These receptors recognize M. tuberculosis and engage the transcription of the *Il1b* gene through mechanisms involving the signaling molecules extracellular signal-regulated kinase (ERK), p38, and Rip2 (56) (Fig. 1). More recently, the transcription of the *Il1b* gene was reported to also be regulated by the glycolytic reprogramming of the macrophage upon M. tuberculosis infection (57, 58), via a mechanism involving the transcription factor hypoxia-inducible factor 1-alpha (HIF-1a) (59) (Fig. 1). Both the blockade of the glycolytic shift with the glucose analogue 2-deoxyglucose and the genetic abrogation of HIF-1α decreased IL-1β production by M. tuberculosis-infected macrophages (57, 58). However, it is important to mention that macrophage metabolic reprogramming induced by M. tuberculosis infection is not completely understood. For example, experiments with killed versus live bacteria yielded different metabolic effects (60), as did experiments using macrophages of different origins (61) and using diverse M. tuberculosis strains (62). Understanding these processes in more detail will better define the link between IL-1β and immunometabolism in the context of TB.

The second step needed for IL-1β production consists of the processing of pro-IL-1β into active IL-1β (Fig. 1). The NLR family pyrin domain containing 3 (NLRP3) inflammasome and its apoptosis-associated speck-like protein containing a CARD (ASC) and CASP1 components (Fig. 1) were implicated in the canonical processing of pro-IL-1β in *in vitro* M. tuberculosis-infected bone marrow-derived macrophages (63–65) and bone marrow-derived dendritic cells (63, 66). The absent in melanoma 2 (AIM2) inflammasome was also shown to contribute to *in vitro* IL-1β production by M. tuberculosis-infected monocytes and macrophages (65, 67, 68). However, *in vivo*, the situation is not so clear. Whereas mice deficient for AIM2 or ASC showed greater susceptibility to infection with M. tuberculosis (67), those deficient for NLRP3 or CASP1 showed little to no difference in their susceptibility compared to wild-type mice and unimpaired IL-1β production (32, 63, 64, 69). The fact that the absence of NLRP3 or CASP1 did not affect IL-1β production hints at the existence of noncanonical mechanisms of IL-1β processing, which may operate *in vivo* independently of NLRP3. These mechanisms may comprise other CASPs, matrix metalloproteases, chymases, elastases, and cathepsins (Fig. 1), all previously proposed as alternatives to CASP1 for IL-1β processing (5). Indeed, the induction and maturation of IL-1β in dendritic cells infected with M. tuberculosis have been described downstream of Dectin-1 activation through a noncanonical CASP8-dependent inflammasome (70). Another possibility, not yet proven in M. tuberculosis infection, is the existence of a two-cell inflammasome-independent model, where invariant natural killer cells instruct antigen-presenting cells to secrete IL-1β via CASP8 activation (71).

**FIG 2:**
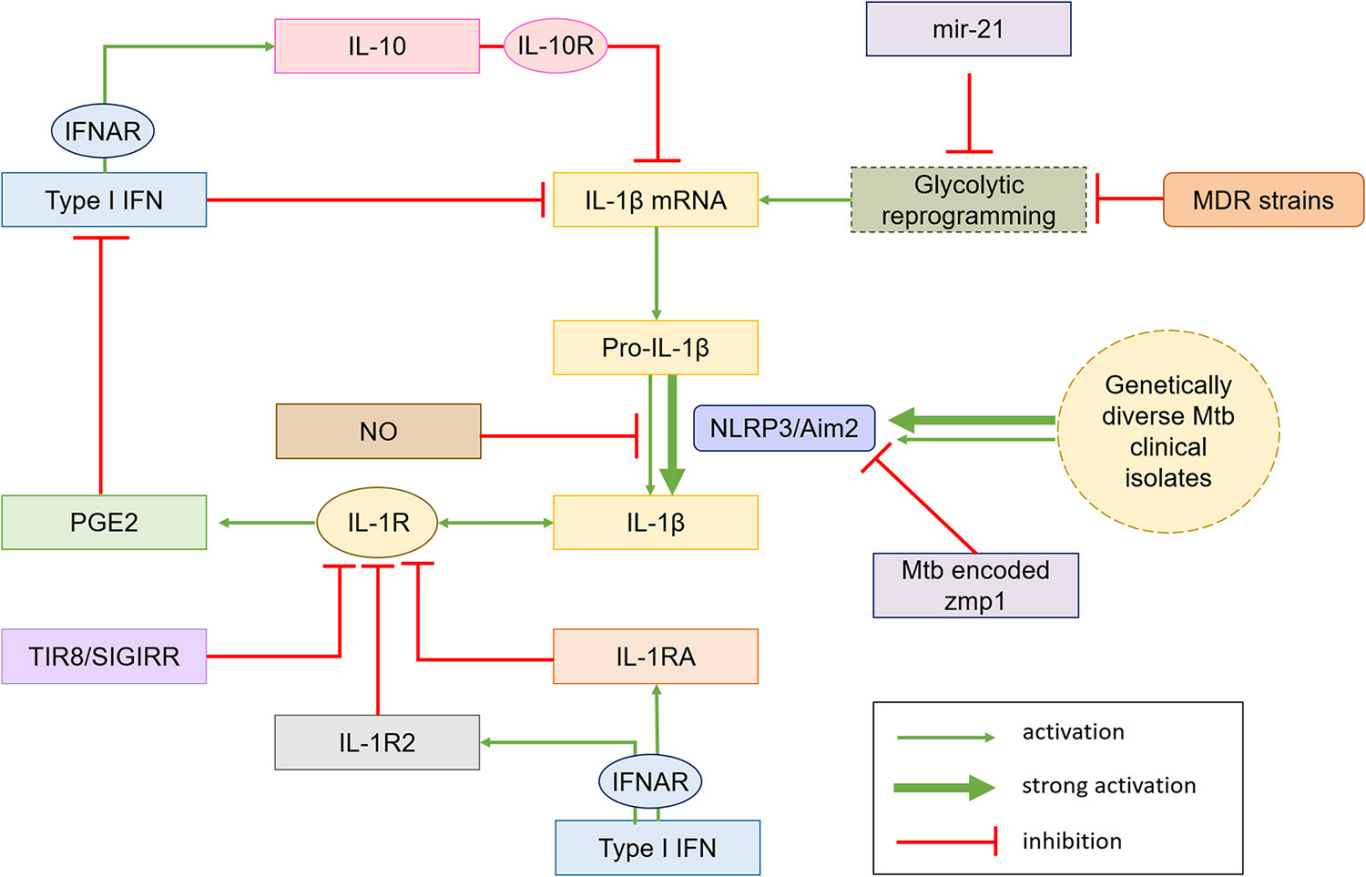
Mechanisms of host and pathogen cross-regulation of IL-1 during M. tuberculosis infection. On the host side, type I IFNs are major regulators of IL-1 responses, acting directly by inhibiting the transcription of pro-IL-1β or indirectly by inducing IL-10, which downregulates the expression of pro-IL-1β, or upregulating IL-1R2 and IL-1RA. TIR8/SIGIRR also blocks IL-1R responses. PGE_2_ induced by IL-1β counterregulates the action of type I IFN by blocking its transcription. At the posttranscriptional level, NO regulates the production of IL-1 by inhibiting the NLRP3 inflammasome. On the pathogen side, M. tuberculosis (*Mtb*) has evolved multiple mechanisms to downregulate IL-1β, from blocking macrophage glycolytic reprogramming to blocking the inflammasome. Genetically diverse clinical isolates of M. tuberculosis have been shown to modulate the production of IL-1β via impacting inflammasome activity. MDR, multidrug resistant.

Several mechanisms for IL-1 cross-regulation have been reported in the context of M. tuberculosis infection (Fig. 2), affirming the importance of maintaining a balance in IL-1 activity. This cross-regulation may be achieved within the IL-1 family itself, through the binding of IL-1RA to IL-1R1 or of IL-1R2 to IL-1 (Fig. 2), both of which prevent the cell signaling response to IL-1, thus limiting its effects (72). Another IL-1R family member with a role in dampening inflammation and tissue damage in M. tuberculosis infection is toll/interleukin-1 receptor 8/single Ig IL-1-related receptor (TIR8/SIGIRR) (Fig. 2), a negative regulator of TLR/IL-1R signaling (73). In its absence, M. tuberculosis-infected mice succumb prematurely and exhibit massive liver necrosis as well as increased levels of IL-1β and TNF-α in lung mononuclear cells and serum (73).

Although IL-1RA is also expressed during TB, therefore restraining the IL-1β-induced response, the most notable cross-regulatory mechanism in the context of M. tuberculosis infection is possibly the interplay established between IL-1β and type I interferons (IFNs) (74) (Fig. 2). M. tuberculosis induces host production of type I IFNs via different processes, from the activation of TLR4 (75, 76) to the activation of intracellular PRRs such as nucleotide-binding oligomerization domain containing 2 (NOD2) (77), cyclic GMP-AMP synthase (cGAS) (78, 79), and retinoic acid-inducible gene I/mitochondrial antiviral signaling protein (RIG-I/MAVS) (78). Type I IFNs directly downregulate the transcription of IL-1β (Fig. 2) in human macrophages infected with M. tuberculosis (80). Molecular signals that negatively regulate type I IFNs in M. tuberculosis-infected macrophages, such as tumor progression locus 2 (Tpl2), were shown to positively regulate IL-1β (81). Furthermore, the production of IL-1 by murine inflammatory monocytes, macrophages, and dendritic cells during mycobacterial infection was suppressed by type I IFNs *in vivo* (16). Interestingly, this activity of type I IFNs in regulating IL-1 *in vivo* is not entirely direct, instead being mostly dependent on the induction of the anti-inflammatory cytokine IL-10 (16) (Fig. 2). Moreover, type I IFNs also upregulate IL-1RA and IL-1R2 (Fig. 2), thus again indirectly inhibiting IL-1 (16, 31, 82). Interestingly, in turn, IL-1 produced during M. tuberculosis infection limits the production of type I IFNs at the translational and transcriptional levels via the induction of PGE_2_ (Fig. 2) in myeloid cells, which directly reduced type I IFN-driven bacterial proliferation and mortality (31). Furthermore, an AIM2–IL-1β signaling pathway has been reported to downregulate the production of *Ifn* genes by inhibiting the STING/TBK1 pathway (83). The cross talk between type I IFNs and IL-1 in TB is likely implicated in the detrimental effects of the former, as illustrated by the observation that the highly susceptible phenotype of IL-1R-deficient mice is abrogated in mice deficient for both IL-1 and IFN receptors (31).

In addition to type I IFN, other immune mediators have been shown to cross-regulate IL-1 responses (Fig. 2). T-cell-derived IFN-γ downregulates the production of IL-1 although only on monocytes and macrophages, implying that IL-1 production may be differentially regulated within different myeloid subsets by different IFNs (16). Furthermore, a role for nitric oxide (NO) in regulating the production of IL-1 (Fig. 2) has been proposed, via nitrosylation and subsequent inhibition of the NLRP3 inflammasome (84). At the functional level, NO halts the neutrophil recruitment cascade driven by IL-1, which, if uncontrolled, becomes detrimental to the host (85). Other molecules that might also intervene in similar processes of inflammatory regulation are reactive oxygen species (ROS), as demonstrated by increased IL-1β production and uncontrolled inflammation in the lungs of NADPH-deficient mice infected with Mycobacterium marinum (86). However, it is important to note that the IL-1 signaling cascade is necessary for the production of ROS and subsequent pathogen control (29), illustrating the importance of regulating IL-1-driven inflammation so that it does not become disadvantageous for the host.

## MANIPULATION OF IL-1 RESPONSES BY M. TUBERCULOSIS

A competent bacterial ESX-1 secretion system is a determinant for the induction of IL-1 in M. tuberculosis-infected cells (79, 87). This system, encoded by genes belonging to the region of difference 1 (RD1) locus, is a virulence factor present in virulent mycobacteria (e.g., M. tuberculosis and M. bovis) and necessary for bacterial phagosome evasion (88). Evasion of the phagosome places several bacterial components in the cell cytoplasm, notably ESAT-6, which has been described to activate the NLRP3 inflammasome (89, 90) and the canonical CASP1 processing of IL-1β through a mechanism that involves the induction of potassium ion efflux in infected phagocytes (87, 91). Both RD1- and ESAT6-deficient M. tuberculosis mutants fail to induce the production of IL-1β (and IL-18) in infected macrophages (87, 91). That a virulence factor contributes to the activation of NLRP3 may seem counterintuitive. However, the ESX-1 secretion system also mediates the export of bacterial DNA and RNA from the phagosome into the cell cytosol, thus triggering the production of type I IFNs through cGAS and RIG-I recognition, respectively (78, 79). Thus, the bacterial mechanisms leading to IL-1 production potentiate, at the same time, the synthesis of type I IFN. The pathogen may be able to take advantage of this cross-regulation by manipulating IL-1 levels, ensuring its survival and progression of disease (79, 80, 92). Moreover, NLRP3 activation may contribute to necrotic cell death, favoring further bacterial escape from the phagosome (93), a process that also involves the TLR2-MyD88 pathway (90, 94). Considering the links between IL-1, TLR, and NLRP3, it is tempting to speculate that IL-1 itself may play a part in this subversion mechanism. M. tuberculosis also upregulates the production of LXA4 by human macrophages, which in turn downregulates the synthesis of PGE_2_ and promotes necrotic cell death, favoring bacterial dissemination (50). Given that IL-1 is necessary for the upstream production of PGE_2_ (31), one could hypothesize that IL-1 manipulation by M. tuberculosis could also be related to the outcome of cell death and consequent bacterial dissemination.

Several mechanisms interfering with inflammasome activation, and thus ultimately modulating the secretion of IL-1β, have been described for M. tuberculosis (Fig. 2). The M. tuberculosis protein encoded by the *zmp1* gene was found to prevent inflammasome activation and IL-1β production (95) (Fig. 2). Mice infected with bacteria lacking *zmp1* displayed higher levels of production of IL-1β, lower bacterial burdens in the lungs, and enhanced bacterial clearance, resulting in a positive outcome of the disease (95). More recently, differential induction of IL-1β by genetically distinct M. tuberculosis clinical isolates was found to be associated with disease severity (65). Clinical isolates recovered from patients with mild TB disease consistently induced high-level secretion of IL-1β in human PBMCs, THP-1 cells, and mouse bone marrow-derived macrophages, which was related to stronger activation of the inflammasome (65) (Fig. 2). Furthermore, M. tuberculosis clinical isolates of the modern lineage 4 were shown to induce a higher-cytokine-production profile, including IL-1β, than those from ancient lineages such as lineage 1 and lineage 5 (96). Of note, the modulation of IL-1β production by isolates of M. tuberculosis may also occur through hitherto-unknown mechanisms (97, 98).

Another potential mechanism used by M. tuberculosis to regulate the production of IL-1β relates to the modulation of the metabolic reprogramming of phagocytic cells (99) (Fig. 2). As discussed above, M. tuberculosis infection drives a metabolic shift in the macrophage toward glycolysis, which is linked to IL-1β production (58). By upregulating the host production of microRNA 21 (miR-21) (Fig. 2), M. tuberculosis interferes with *PFK-M*, preventing the glycolytic shift and decreasing the production of IL-1β, a process that culminates in enhanced bacterial replication within macrophages (57). Interestingly, IFN-γ was shown to reverse this mechanism and to augment the production of proinflammatory cytokines like IL-1β (57). These results may seem in contrast to others mentioned above, where IFN-γ suppressed the production of IL-1β (16), but these differences could be explained by the specific cellular context as well as the use of different M. tuberculosis strains in these studies. Indeed, bacterial diversity was shown to impact both the metabolic modulation of the macrophage (99) and the production of IL-1β (65, 96–98). Another example of cross-modulation between metabolism and IL-1 is seen in macrophages infected with M. tuberculosis strains carrying a rifampicin drug resistance mutation (Fig. 2), which altered the expression of M. tuberculosis lipid wall components, impacting macrophage metabolic reprogramming, bypassing IL-1 signaling, driving the induction of IFN-β, and inhibiting glycolysis (62). Given that during M. tuberculosis infection *in vivo*, macrophages of different ontogenies were shown to follow distinct metabolic reprogramming (61), it will be of interest to understand how this may correlate with IL-1β regulation by alveolar versus interstitial macrophages.

## THE CLINICAL POTENTIAL OF IL-1 IN TB

Given the role of IL-1 in TB pathogenesis, the development of IL-1-based novel TB interventions is an exciting possibility. A challenge will be to pinpoint the individuals who will benefit more from such therapies, namely, whether they are latently infected or have active TB, with or without comorbidities. Currently, commonly employed IL-1-based therapies are aimed at treating inflammation, based on blocking the effect of IL-1 signaling. These include the administration of the synthetic IL-1RA anakinra, soluble decoy receptors for IL-1, or anti-IL-1 neutralizing antibodies (11). Since immune-suppressive treatments, including anakinra (12–14), are associated with an increased risk of the development of TB, it is tempting to speculate that enhancing IL-1 signaling without causing excessive inflammation may be helpful in protecting the host from TB disease progression. A host-directed therapy (HDT) that is intrinsically linked to IL-1 is the targeting of the host eicosanoid network (31). As mentioned above, PGE_2_ is a downstream product of IL-1R signaling that promotes apoptotic cell death and limits bacterial proliferation (31, 50). Since type I IFNs downregulate the production of PGE_2_, balancing the immune response between IL-1 and type I IFNs is a promising HDT to manipulate PGE_2_ production, thus favoring bacterial containment (31). Another interesting HDT based on IL-1 in TB relates to the potential role of IL-1 in trained immunity (100). A recent study has shown that mice trained with β-glucan showed enhanced protection against M. tuberculosis infection, a phenotype that depended on a competent IL-1R and that was associated with the expansion of hematopoietic stem and progenitor cells in the bone marrow and increased myelopoiesis (101). Exploring a possible role for IL-1 in myelopoiesis and the development of trained immunity during TB may be an important HDT to further pursue in the future. Importantly, excessive IL-1 is detrimental in TB, so the therapeutic control of this cytokine at certain stages of infection, and possibly anatomical locations, may benefit the host. A recent study showed that the side effects of linezolid, a potent antibiotic effective in TB treatment, were abrogated upon IL-1 blockade, without affecting host resistance to TB, in mice and nonhuman primates (42). Also, a recent study suggested that for M. tuberculosis strains that elicit a high IL-1β response, NLRP3 inflammasome inhibition may reduce bacterial survival in macrophages (98). However, increased amounts of IL-1 production elicited by clinical isolates of M. tuberculosis may reflect better outcomes of TB severity (65). Altogether, the modulation of IL-1 responses in TB requires a detailed understanding of the mechanisms through which IL-1 affords protection or pathology and will also likely need to take into consideration the levels of IL-1 induction provided by different host-M. tuberculosis pairs, exposed to different lifestyles and epidemiological conditions.

While IL-1-based HDTs may hold promise, there is also evidence that the IL-1 family could be useful as a biomarker in TB. A larger amount of IL-1β produced *ex vivo* by M. tuberculosis-stimulated macrophages from individuals with latent TB than from individuals with active TB has been reported (102). The high-level production of IL-1β by monocytes and dendritic cells upon TLR stimulation was associated with a lower incidence of TB recurrence, while IL-1β production upon stimulation with M. bovis BCG was correlated with a higher risk of TB relapse (103). Furthermore, higher IL-1β levels measured in the peripheral blood were associated with higher bacterial loads in the sputum, granuloma cavitation, and elevated disease severity in TB patients, which were decreased upon TB treatment (104, 105). IL-1α, in combination with epidermal growth factor (EGF), sCD40L, vascular endothelial growth factor (VEGF), and transforming growth factor α (TGF-α) produced upon antigen stimulation of whole blood, was also used to distinguish between active and latent TB cases (106). Finally, some studies place IL-1RA measured in antigen-stimulated whole blood and QuantiFERON assays as a potential marker to distinguish cases of active versus latent infections (107–110). IL-1RA was also reported as a potential surrogate marker to monitor the efficacy of TB treatment in HIV patients diagnosed with TB (111). A major limitation of these studies, and indeed of using IL-1 family members as potential biomarkers of TB, relies on the difficulty in drawing the threshold of their production relevant to each clinical scenario, an issue that warrants further investigation.

## CONCLUSIONS AND OUTSTANDING QUESTIONS

Compelling evidence positions IL-1 as a fundamental player in the immune response against TB. The lack of functional IL-1R signaling results in an inability to control the infection, as seen at a cellular level (28, 31, 49, 50) or in the complex setting of *in vivo* infections (16, 30, 32–34, 38, 112). However, it is also evident that enhanced IL-1 responses lead to severe inflammation and tissue damage, thus also compromising host resistance to TB (42, 73, 98). Importantly, there are still important gaps in our knowledge on the precise role of IL-1 and its full contribution to the immune response in TB, specifically in the human context, as the majority of studies are performed *in vitro* and *in vivo* with animal models. Some outstanding questions persisting in this field are as follows:

- How does IL-1 modulate susceptibility or resistance to infection in humans?
- What is the role of less-studied IL-1 family members in TB?
- What are the hematopoietic and nonhematopoietic cellular targets of IL-1 during the course of M. tuberculosis infection?
- Is the role of IL-1 in TB dependent on the anatomical location, e.g. alveolar space versus lung interstitium or bone marrow versus lung?
- What is the role of IL-1 during early versus late stages of infection, and how does that affect the outcome of TB?
- Which molecular mechanisms operate *in vivo* to regulate the production of IL-1 during M. tuberculosis infection?
- Can we explore the natural genetic diversity of M. tuberculosis to uncover novel strategies directed at modulating host IL-1 responses?

All in all, given the enormous complexity of TB, developing and translating IL-1-based therapies may seem a herculean task. However, the advances discussed here place us on the right path to identify and understand the key points involved in this complexity. This in turn is critical to lead to the right questions and, most importantly, design both meaningful clinical studies and appropriate experiments, namely, to model human TB to animal models and back. These concerted efforts will undoubtedly move the field toward the clinical application of IL-1 in TB.
